# The characteristics of drug information inquiries in an Ethiopian university hospital: A two-year observational study

**DOI:** 10.1038/s41598-019-50204-1

**Published:** 2019-09-25

**Authors:** Yonas Getaye Tefera, Begashaw Melaku Gebresillassie, Asnakew Achaw Ayele, Yared Belete Belay, Yohannes Kelifa Emiru

**Affiliations:** 1 Department of Clinical pharmacy, School of Pharmacy, College of Medicine and Health Sciences, University of Gondar, P.O. Box: 196, Gondar, Ethiopia; 2 Department of pharmacognosy, School of Pharmacy, College of Medicine and Health Sciences, University of Gondar, P.O. Box: 196, Gondar, Ethiopia; 3 Department of pharmaceutics, unit of Social Pharmacy, Mekelle University, Mekelle, Ethiopia

**Keywords:** Drug regulation, Health services

## Abstract

The types of drug-related information request from patients and health professionals, the extent of inquiry and capability of existing drug information centers are seldom studied in Ethiopia. This study aimed to identify the types and potential areas of drug information inquiry at the Drug Information Center (DIC) of Gondar University specialized Hospital (GUSH), Ethiopia. An observational study was employed. The drug information query was collected by distributing the drug information queries in different hospital units through two batches of graduating undergraduate pharmacy students. Descriptive statistics used to describe, characterize and classify drug related queries. Binary logistic regression test was employed to identify predictor variables to type of drug information query. A total of 781 drug related queries were collected and 697 were included in the final analysis. Near to half (45.3%) of queries comes from the pharmacists followed by general practitioners (11.3%) and nurses (10.2%). Slightly greater than half of the queries (51.9%) were focused on therapeutic information. 39.6% of drug related queries related to infectious disease case scenarios, followed by cardiovascular cases in 21.3% of queries. More than half of (53.9%) and nearly one in five (19.4%) of the queries took 5 to 30 minutes and 30 minutes to 1 hour of literature searching to answer, respectively. Pharmacists (with odds ratio of 2.474(95% CI (1.373-4.458)) and patients (with odds ratio of 4.121(1.403–12.105)) ask patient-specific questions in their drug related queries higher than other group of health professionals. Pharmacists are the primary drug information users and frequent drug related information inquirers at the DIC. Most of the queries targeted therapeutic indications, adverse drug events, infectious or cardiovascular disease related requests. This is imperative that drug information services can assist the growing role of pharmacists in addressing the patient specific drug related needs.

## Introduction

Drug information is needed to assist various clinical decisions and will help to utilize well supported evidences for better patient care and clinical outcomes. Medicine information is encompassing information focused at healthcare professionals, patients and consumers with the primary aim of educating and ensuring the quality, safety, effectiveness and appropriate utilization of medicines^1^. From the establishment of the first drug information center at Kentucky, USA in 1962, drug information services expanded throughout the world with astounding technological improvement^2^. In Ethiopia, establishment of drug information services in health facilities is a decade old, the first drug information center (DIC) is launched in 2009 at Tikur Anbesa Specialized hospital which is affiliated to college of health sciences of Addis Ababa University^3^. Though health facilities that have drug information services is barely distributed in the country, health facilities with drug information services are also not functioning in full capacity. The other drug information center of the country is established in 2011 at Gondar university specialized hospital (GUSH) in the northwestern Ethiopia. It is among the pioneer four drug information centers established in the country which are also affiliated with universities and teaching hospitals^4^.

Drug information services refers to a service that encompasses the activities of specially trained individuals to provide accurate, unbiased, factual drug information, primarily in response to patient-oriented problems^5,6^. These days, there has been a rapid expansion in the number and diversity of pharmaceutical market available, complexity of medication therapy, and the need for evidence-based patient treatment. It all leads to increased demand of consultation regarding therapeutic indication, medicine selection, comparative effectiveness and safety, proper medication use with current evidences and updated literatures. With the existence of abundant literatures as source of drug information, quality is very crucial aspect to utilize the evidence in the clinical setting with paramount confidence^7^. The quality of drug information is determined by its accessibility, reliability, completeness, and applicability to the most of practice perspectives^8^. Therefore, trained professionals needed to ensure the information quality and to make decisions on resource selection, extraction, analysis of drug information and apply the evidences in context of the real practice from the large pool of available data. With this regard, drug information centers have tremendous role as source of applicable and reliable drug information for healthcare settings with professional information filtering and censorship through applying Watanabe *et al*. modified systematic approaches of to reply drug information queries^9,10^.

The current healthcare practice demands shared clinical decisions of all involved healthcare practitioners and patients. This might put multiple healthcare discipline professionals and patients in mutual collaboration, need of evidence-based information and medicine. Queries concerning at patients’ clinical management arise in daily healthcare practice, with 0.16 to 1.27 questions per patient depending on the methodology used and the clinical setting^11^. Enabling patient’s involvement in their disease management and clinical decisions requires unreserved informed medical consultation and adequate provision of drug information. This will help in improved patient adherence and health outcome aided by the receiving of relevant and tailored medicines information which has been developed to high quality standards^1^.

## Aim of the Study

The drug related information demand, pattern of enquiry, functionality and improvement of existing drug information centers are seldom studied in Ethiopia. Hence, such kind of study on drug information queries were supposed to assess the type of requests and medicine information seeking behaviors in the healthcare setup. This is the prime study to evaluate drug related queries prospectively in Ethiopia with the aim of identifying the types and potential areas of drug information enquiry in Ethiopian healthcare setting from health care providers and patient’s perspectives.

## Methods

### Study setting and design

Gondar University Specialized Hospital (GUSH) is a teaching tertiary hospital serves for 7 million people in the catchment area of northwest Ethiopia and comprised more than 1000 beds for admitted patients. The hospital has medical, surgical, pediatrics, gynecology-obstetrics, oncology, emergency, ambulatory, dental, ophthalmic, psychiatric departments and various pharmacy units. The drug information center (DIC) at GUSH is staffed with one trained full-time drug information pharmacist and a library consisting of textbooks, guidelines and equipped with computers and internet facilities along with databases such as Up-to-date. It is also a practice site for the graduating class pharmacy students every year as part of the clinical clerkship attachment. The DIC was both pharmacist and student-run service since it is practical site for pharmacy students for their DIC clinical clerkship. They were providing drug information service for clients under the supervision of the drug information pharmacist and the clinical preceptor.

An observational study was employed from January, 2016 to December, 2017 at DIC of GUSH. The study query was collected through 2 batches of graduating class of undergraduate pharmacy students as partial fulfilment their drug information clerkship evaluation. This clerkship attachment was rotation of every 2 weeks for group of 5 students throughout the academic year except July and August which was student vacation. 63 students in year 2016 and 41 students in 2017 were responsible to collect drug related queries in their DIC attachment. Every student is supposed to collect 8 queries in their 2-week attachment at drug information center by distributing the drug information query forms in different hospital wards, inpatients, physician morning sessions, medical rounds and pharmacies. Then the students are evaluating the query form through systematic modified approach and provides the appropriate response as wanted via verbal, written, supplying literature or referring under the supervision of drug information pharmacist and approval of clinical pharmacy preceptors who is mentoring at DIC.

### Data collection, quality control and management

The students got appropriate orientation and guidance on the first two days of their DIC attachment about drug information query collection, search strategies, literature evaluation and replying process through modified systematic approach of drug related queries as it is one of their robust academic evaluation processes in DIC. They are not informed about it would use for further research purpose beyond the academic evaluation and the routine practice. The students were not aware of the ongoing observations in order to prevent bias towards the outcome under investigation. The principal investigator did not interfere in the reference selection, way of responding and other drug information service activities except the mentorship and supervision of the whole process during drug information service provision. The principal investigator only observes the usual query collection, flow of drug information, provides appropriate orientation and academic guidance as per the request.

Close supervision was done on daily basis regarding students’ query collection and information provision process. Quality assessment was done for the completeness and originalities of each query. The responses provided checked and ensured whether it reached for the requester. All incomplete query forms which missed two or more variables were not included to this study.

The drug information query form was developed by the team of pharmacists and lecturers from the drug information center and school of pharmacy, University of Gondar. The query form is used for the routine drug related request service comprised with various components of the socio-demographic characteristics of the requestors, the relevant specific patient clinical parameters (History, medication regimen, diagnosis, pertinent laboratory findings are among others), the actual drug information request, classification of the request, references consulted, the type of query and researching hour was included

### Data analysis

Statistical analyses were performed using the Statistical Package of the Social Sciences software, version 20.0 (SPSS Inc., Chicago, IL, USA). Descriptive statistics used to describe, characterize and classify queries with various elements. Binary logistic regression test was employed to identify predictor variables to type of queries in terms of patient specificity. P-value less than 0.05 and 95% confidence interval (CI) used as cut off points for determining statistical significance of associations among different variables.

### Ethics approval

The study was initiated after obtaining ethical clearance approval from ethical review committee of the school of pharmacy, University of Gondar. The ethical approval was given in accordance with the World medical association declaration of Helsinki- Ethical principles for medical research involving human subjects and the 2014 national research ethics review guideline adoption to institutional review boards of Ethiopia. No personal identifiers were included in the study; socio-demographics of requestors and their questions kept anonymous. Written informed consent was not requested prior to the data collection from the drug related query requestors since it could affect the outcome under investigation. But at later stage of the data collection, informed verbal consent was obtained to use the data provided under observation.

## Result

### Sociodemographic and general query characteristics

A total of 781 drug information queries collected in the 2 years period and 697 included in the final analysis. Mean age and weight of the patients in the drug related query was 31.16 ± 20.12 years, 45.16 ± 21.4 kg respectively. Near to half (45.3%) of queries come from the pharmacists followed by general practitioners (11.3%) and nurses (10.2%). Nearly all (95.8%) of the queries were collected by visit query distribution and the mode of reply was oral in most (83.1%) of the queries. Slightly greater than half (51.9%) of the queries were focused on the therapeutic information and followed by the request of adverse drug event information (11%), pharmacology (8.8%) and dose (5.9%) while the least required information’s were on price (0.3%), quality (0.3%), bioequivalence (0.4%) and pharmaceutics questions (0.4%) (Table 1).

**Table 1:** Socio demographic characteristics of drug information requestors at Gondar University Specialized Hospital, 2018 (N = 697).

Variables	Frequency (%)
Qualification of the requestor	
General Practitioner	79(11.3%)
Specialist Physician	39(5.6%)
Pharmacist	316(45.3%)
Nurse	71(10.2%)
Health officer	47(6.7%)
Patient	23(3.3%)
Caregiver	9(1.3%)
Intern	55(7.9%)
Druggist	4(0.6%)
Other	54(7.7%)
**Method of Receipt of the query**	
Visit	668(95.8%)
Phone	25(3.6%)
E-mail	4(0.6%)
**Mode of reply**	
Oral	579(83.1%)
Written	91(13.1%)
Literature supplied	13(1.8%)
Referred	14(2.0%)
**Classifications of Request by Category**	
Therapy	362(51.9%)
Pregnancy	18(2.6%)
ADR	77(11.0%)
Interaction	41(5.9%)
Quality	2(0.3%)
Pharmaceutical	3(0.4%)
Pharmacology	61(8.8%)
Pharmacokinetics	19(2.7%)
Local/foreign equivalence	3(0.4%)
Availability	14(2.0%)
Price	2(0.3%)
Dose	41(5.9%)
Administration	21(3.0%)
Other	33(4.7%)

In-house databases (40.7%), internet sources (24.4%) and reference books (20.5%) were the three most used source of information consulted to reply drug related queries in the drug information center. More than half of (53.9%) and nearly one in five (19.4%) of the queries took 5 to 30 minutes and 30 minutes to 1 hour of literature searching, respectively. More than half (53.1%) of the queries were patient specific drug related questions. 64.7% of the drug related queries raised in 2016 **(**Table 2**)**.

**Table 2:** Query Characteristics in the drug information request at Gondar University Specialized Hospital; 2018 (N = 697).

Variables	Frequency (%)
**Sex of patients in the Query**	
Male	187(26.8%)
Female	173(24.8%)
Not specified	337(48.4%)
**Patient diagnosis included in the query**	
Yes	502(72%)
No	195(28%)
**Reference Types**	
Reference books	146(20.9%)
Journals	32(4.6%)
In-house database	284(40.7%)
Peer reviewer	3.9%
Internet sources	170(24.4%)
Packaging inserts	4(0.6%)
Other drug information services	17(2.4%)
**Time taken for Researching**	
0–5 mins	103(14.8%)
5–30 mins	375(53.8%)
30 mins – 1 hour	135(19.4%)
1–4 hours	60(8.6%)
4–8 hours	23(3.3%)
**Question type**	
Patient specific Questions	370(53.1%)
General type Questions	327(46.9%)
**Year of Query**	
2016	451(64.7%)
2017	246(35.3%)

### Query classification by patient diagnosis

**Figure 1:**
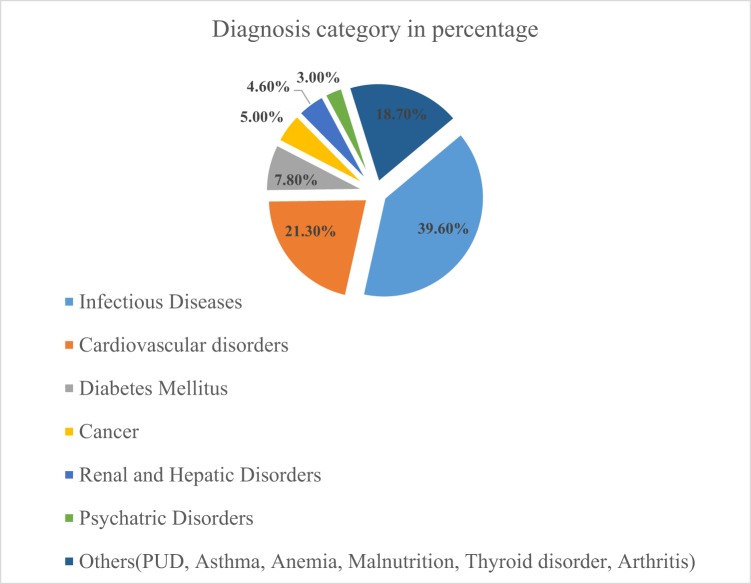
Query classification by the patient diagnosis requested at GUSH DIC, 2018.

More than a third (39.6%) of drug related queries come with infectious disease case scenarios followed by cardiovascular (21.3%) and diabetes mellitus related cases (7.8%). Nearly one in five (18.7%) of the queries were coming from the group of patient cases designated as other which include peptic ulcer disease (PUD), asthma, anemia, malnutrition, thyroid disorders and arthritis (Fig. 1).

### Query classification by drug pharmacologic category

Majority (78%) of the requests coming to the drug information center were only drug related queries in nature. Of which, nearly a quarter (23.5%) of requests information were pertaining to antibiotics, followed by analgesics (8.3%) and steroid (7.9%) related queries (Table 3).

**Table 3:** Query characterization encompassing on different medication classification at the DIC of GUSH, 2018. (N = 697).

Query by Medication categories (n = 544, 78%)	Frequency	Percentage from total query	Percentage from medication related query
Antibiotics	128	18.4%	23.5%
Antiretrovirals	23	3.3%	4.2%
Antimalarials	18	2.6%	3.3%
Antihelminth	14	2.0%	2.6%
Anti TB	19	2.7%	3.5%
B-blockers	16	2.3%	2.9%
Diuretics	19	2.7%	3.5%
ACE inhibitors	13	1.9%	2.4%
CCB	10	1.4%	1.8%
Hypoglycemic agents	25	3.6%	4.6%
Analgesics (opioids and NSAIDS)	45	6.5%	8.3%
Steroids	43	6.2%	7.9%
Anticoagulants + antiplatelets	24	3.4%	4.4%
PPIs and acid suppressants	20	2.9%	3.7%
Antidepressants	8	1.1%	1.5%
Chemotherapy	13	1.9%	2.4%
Vitamins and supplements	20	2.9%	3.7%
Others group of Medications	86	12.3%	15.8%
**Total**	**544**	**78.0%**	**100.0%**
Other than drug related Queries	153	22.0%	
**Total**	**697**	**100.0%**	

TB- Tuberculosis, B-Blocker -Beta blocker, CCB- Calcium Channel Blockers.

NSAIDS- Non-Steroidal Anti-Inflammatory Drugs.

### DIC reference utilization and resource category

Most (82.5%) of the queries replied through using tertiary drug information resources, of which Up-to-date was the single foremost used drug information source to respond in 284 (40.7%) of the total queries coming to drug information center. 3.4% of the drug related queries answered by researching primary sources which were original articles while 0.7% of queries are replied from PubMed (secondary literature) (Table 4).

**Table 4:** Reference category and specific drug information resources consulted to respond drug related queries at GUSH DIC, 2018 (N = 697).

Reference category	Type of references used to reply queries	Frequency	Percentage (%)
Tertiary Resources (n = 575, 82.5%)	Leaflet/package inserts	4	0.57%
	Good man and Gilman’s pharmacology, 12^th^ edition	7	1%
	Katzung pharmacology, 10^th^ edition	10	1.43%
	Nelson text book of pediatrics, 19^th^ edition	10	1.43%
	Standard treatment guideline of Ethiopia, 2014 edition	11	1.57%
	Harrison Principle of Internal medicine, 19^th^ edition	20	2.87%
	Kodak -Kimble & young’s applied therapeutics, 10^th^ edition	30	4.3%
	Dipiro Pathophysiologic approach of Pharmacotherapy, 8^th^ edition	33	4.73%
	Unspecified Internet sites	82	11.76%
	Medscape	84	12.05%
	Up-to-date version 21.2	284	40.7%
Primary resources (n = 24, 3.4%)	Original articles	24	3.4%
Secondary resource (n = 5, 0.7%)	PubMed	5	0.7%
	Peer review and Asking	27	3.9%
	Reference specifically not mentioned	66	9.5%
	Total	697	100%

### Predictors to patient specificity of queries

Qualification was the only predictor variable associated to ask either patient specific case queries or general type of questions. From the requestors, being a pharmacist and patient were associated with higher likelihood to ask patient specific drug related queries with Crude odds ratio of 95% CI, 2.474(1.373, 4.458) and 4.121(1.403–12.105), respectively. Pharmacists and patients ask patient specific questions in their drug related queries 2.5 and 4.12 times of others, respectively; as other group of professionals which held as a reference for comparison (Table 5).

**Table 5:** Binary logistic regression test (crude odds ratio) for qualification of the requester predicted type of question asked either Patient specific and general type questions.

	Question Type		B	S.E.	Wald	df	Sig.	Exp(B)	95% CI. for EXP(B)	
	Patient specific (n = 370)	General (n = 327)							Lower	Upper
**Qualification**					37.113	9	**0**.**000**			
General Practitioner	35	44	0.146	0.358	0.166	1	0.684	1.157	0.574	2.333
Specialist Physician	15	24	−0.095	0.430	0.049	1	0.825	0.909	0.391	2.112
Pharmacist	199	117	0.906	0.300	9.089	1	**0**.**003**	2.474	1.373	4.458
Nurse	26	45	−0.174	0.371	0.220	1	0.639	0.840	0.406	1.738
Health officer	18	29	−0.102	0.408	0.063	1	0.902	0.903	0.406	2.010
Patient	17	6	1.416	0.550	6.636	1	**0**.**010**	4.121	1.403	12.105
Caregiver	5	4	0.598	0.726	0.679	1	0.410	1.818	0.438	7.540
Intern	30	25	0.557	0.387	2.068	1	0.150	1.745	0.817	3.729
druggist	3	1	1.473	1.187	1.539	1	0.215	4.364	0.426	44.731
Constant	22	32	−0.375	0.277	1.830	1	0.176	0.687		

CI – Confidence Interval.

## Discussion

The drug information center training and service provision could be an effective model for teaching evidence-based healthcare to pharmacy students to equip them with the necessary practical skills and knowledge. Students could feel better prepared for their future role as pharmacists because of the knowledge and skills acquired in the practical drug information services during their internship. In one Brazilian drug information center, the 5-week evidence-based drug information training module enhanced students’ knowledge and skills as well as their ability to apply clinical evidence to actual drug information requests. This learning approach in undergraduate pharmacy education was found to be effective^12^. While at the university of Tennessee in the weekly active learning activities in a Drug information and literature evaluation course, students reported that active-learning strategies contributed to their knowledge of materials covered in the Drug Information and Literature Evaluation^13^.

This observational study identified and characterized the type of drug related inquiries presented in drug information center of Ethiopian university hospital to highlight the practice, need and extent of service provision. Relative majority (45.3%) of the medication related query comes from pharmacists which showed the high demand of drug information were in the pharmacy side. This might indicate their good awareness of the available drug information services in the setting. On the other end, clinical pharmacy service is introduced in the hospital wards which might laid pharmacists in forefront of drug information consultation and in turn increase their drug related enquiry^10,14^. It is quite less than from the multicenter study conducted in Ethiopia which stated 63.5% of the drug related queries came from Pharmacists^15^ In contrary to one study in Ethiopia, other studies in India and Uganda revealed that physicians found to be the frequent drug information users than pharmacists^5,16–18^. While patient drug related queries to the drug information center is minimal in present study which is by far lower than the Iranian patients’ drug information use^19^. Lower utilization of the DICs in Ethiopia by the patients might be due to poor awareness to existing drug information services, lower literacy level and skimpy health seeking behavior. This might underestimate the patient’s demand of drug information services. But the anticipated patient drug related necessity is broader in scope and heterogeneous in its type which will need unreserved effort and demonstrative promotions^15,18,20,21^.

Almost all requests were obtained directly by visit and most of them were replied orally. This might be the students who visit in the hospital wards, medical mornings and different pharmacy units would probably enable healthcare workers to submit their drug related query while the students proactively invited them for any drug related request. Though it was student facilitated query, it could tell workplace demands of drug information request was high which might suggest periodic work place visit. Drug information pharmacists should visit different units with in the hospital apart from DIC to collect drug related queries and meet the drug information demand of healthcare professionals. Besides to these, low percentage of requests coming through phone and email in this study showed that quite extensive procedures might discouraged them to meet the drug related needs via such long process. Perhaps, it could be justified since there were plenty of options to get drug information with in shorter period of time through personal digital assistances such as mobile apps, internet and other online sources^22^.

Tertiary drug information sources were used as the most utilized references to respond in the most (82.5%) of drug related queries. Of which in-house databases like Up-to-date, internet sites and textbooks were the main references used during the period of this study. Up-to-date version 21.2 is the most extensively used resource in the 40.7% of queries while other Indian and Iranian studies showed Micromedex is broadly utilized source of information in 72% and 52.48%, of queries, respectively^17,19^. In previous Ethiopian study, Micromedex used to respond in the 19% of queries while Up-to-date version 21.2 used in 15% of drug related queries but in this study Up-to-date use is rise quiet sharply^15^. The Micromedex software license subscription has been used before and expired these days at the DIC of GUSH which might cause the shift to use the more feasible and accessible in-house databases such as Up-to-date and other text books. Even though the type of request determines the kind of reference to be used while responding queries; the concise, general accuracy and simplicity of tertiary resources made them preferable for use. Although the primary resources can provide detail and in-depth information from the clinical trials and original study subjects, their use is not convenient and time friendly as compared with the tertiary resources^10,11^.

Slightly greater than half of the queries were drug therapy related requests which is relatively higher than the multicenter study conducted in Ethiopia that puts drug interaction queries taking the lead^15^. This is quite similar with the Ugandan study that revealed drug queries associated with therapy were the commonest^18^. Nowadays the pharmacist’s role in the clinical patient care is growing which would increase the queries related to therapeutic indications and comparative effectiveness coming to pharmacists. There was slight rise in adverse drug reaction queries which accounted 11% of the queries in the present study. This is somewhat higher than 8.7% of the previous Ethiopian study^15^.

Queries on antibiotics are the frequently requested than any other pharmacological class of medication which agrees with the previous studies in Ethiopia, Uganda and Malaysian public hospitals revealed relative majority of queries are on antimicrobials^15,18,23^. Infectious disease related drug queries were frequently encountered in 39.6% of requests followed by cardiovascular scenarios in 21.3% of queries. These large number of queries related to infectious diseases and antibiotics had been frequent since infectious diseases are rampant and its in the developing countries which Ethiopia belongs to. Despite the greatest infectious disease burden, non-communicable diseases are also on the epidemiological rise and transition which accounted significant requests in the present study^24,25^. Thus, non-communicable disease related drug requests such as cardiovascular and diabetes medication associated queries which followed the antibiotics related queries seems reasonable with existing disease epidemiology. These huge number of infectious disease and antibiotics related queries would indicate the necessity and role of drug information centers as one of the key stakeholders to assure proper antibiotic stewardship program and implementation^26^. Non-steroidal anti-inflammatory drugs and steroid related queries were among the repeatedly encountered requests which might be associated with their high consumption in disease symptom management^27^.

Most of the queries took 5 minutes to 1-hour time to complete the literature searching and responding to requesters. This seems searching strategy was not time intensive as tertiary resources are extensively consulted to reply drug related queries in this study and availability of fast broadband connection would fasten the search process^18,28^. More than half of drug related requests were specifically related to a particular patient scenario which needs rough review of patient medical and medication history to identify the ultimate drug related need. Those reasonably large number of inquiries were patient specific and targeting better patient care. 31.14% of queries in India and 34.3% of drug related enquiries in previous Ethiopian study are aiming better treatment outcomes while in this study queries targeting specific patient scenarios are quite elevated^15,29^. Being pharmacist in profession or a patient is only predicting the patient specificity of queries asked while the rest of variables have no association with the type of questions raised in the query form. Patients supposed to be concerned about their particular disease or drug related entity alone and pharmacists found to be raising patient specific drug requests which is imperative for the growing role of pharmacist in patient care. This might be better supported by the emergence of clinical pharmacy services which strives better patient care in Ethiopia. Post-graduate pharmacy education focused on drug information residency trainings should be emphasized to meet the growing demands of drug related queries in Ethiopia. This could be one of the promising opportunities for pharmacists to render professional services and improve patient care in the clinical setting.

### Limitation of the study

It was proactively initiated student driven request which might not show the actual and genuine demand of the requesters’ intention to ask. Spontaneous queries came once the students promoted and proactively initiated whether they had drug related requests in their workplace. This might have difficulty to infer about the usual drug related information practice and the service utilization. There may be a possibility of student confidence and knowledge bias that could affect the reference selection and researching. Despite this drawback, it will give clue for type of drug information needed from healthcare perspectives in the clinical setting of Ethiopian healthcare facilities.

## Conclusion

Pharmacists were the primary drug information users and frequent drug related information inquirers. Most of the queries targeted therapeutic indications, adverse drug events, infectious and cardiovascular disease related requests. Tertiary information resources such as Up-to-date and text books were extensively consulted to respond the drug related queries. The time spent for researching and synthesize response for the queries was less than one hour for most of the requests. More than half of queries were patient specific drug related requests. Pharmacists and patients were found to be highly associated with patient specific queries which is imperative to the growing role of pharmacists in the patient care and addressing patient specific drug related needs in Ethiopia.

## Supplementary information

Drug query form

## Data Availability

The datasets generated during and/or analyzed during the current study are available from the corresponding author up on reasonable request.
