# Supplementary material for: The characteristics of drug information inquiries in an Ethiopian university hospital: A two-year observational study
Source: Sci Rep. 2019 Sep 25;9:13835. doi: 10.1038/s41598-019-50204-1 (PMC6761201; doi:10.1038/s41598-019-50204-1)
Supplement: Supplementary file 1 — Drug query form [file 41598_2019_50204_MOESM1_ESM.pdf]

The Characteristics of Drug Information Inquiries in An Ethiopian University Hospital: A Two Year Prospective Observational Study

Yonas Getaye Tefera[1*], Begashaw Melaku Gebresillassie, Asnakew Achaw Ayele[1], Yared Belete Belay[3], Yohannes kelifa Emiru[2]

1. Department of Clinical pharmacy, School of Pharmacy, College of Medicine and Health Sciences, University of Gondar, P.O. Box: 196, Gondar, Ethiopia.

2. Department of pharmacognosy, School of Pharmacy, College of Medicine and Health Sciences, University of Gondar, P.O. Box: 196, Gondar, Ethiopia.

3. Department of pharmaceutics, unit of Social Pharmacy, Mekelle University, Mekelle, Ethiopia.

***Yonas Getaye Tefera** (Corresponding Author), Bpharm, Msc. Lecturer, Department of Clinical Pharmacy, School of Pharmacy, College of Medicine and Health Sciences, University of Gondar, P.O. Box: 196, Gondar, Ethiopia

Tel: +251-9159494, E-mail address: yonas1get@gmail.com

ANNEX

Gondar University Specialized Hospital-Drug Information Center

Query Form

Name of enquirer

Region

Address

Physical address

Tel No.

E-mail

Fax

Method of Receipt

Visit

Phone

E-mail

Fax/letter

Reference No. __________

Date________________

Qualification

GP

Specialist

Pharmacist

Nurse

Health Officer

Patient

Caregiver

Other

Drug/Product

Indication

Other drug therapy

Time

_____________ a.m.

_____________ p.m.

References

____

____

____

____

Query type

____

Patient specific Question

____

General Question

____

Classification

Therapy

Pregnancy

Lactation

ADR

Interaction

Quality

Pharmaceutical

Pharmacology

Pharmacokinetics

Local/Foreign equiv.

Availability

Price

Dose

Administration

Other

Patient data

Age Wt M/F

Diagnosis

Reference sources

<table>
<tr><td>History</td><td rowspan="3">Reply
Oral
Written
Literature supplied
Referred</td><td rowspan="2">Reference books
Journals
In-house database
Peer reviewer
Internet sites
Package inserts
Other drug info service
Other</td></tr>
<tr><td></td></tr>
<tr><td></td><td>Name/Initials</td></tr>
<tr><td colspan="2">Question</td><td>Follow up</td></tr>
<tr><td colspan="2"></td><td rowspan="2">Key words</td></tr>
<tr><td colspan="2">Answer</td></tr>
<tr><td colspan="2"></td><td rowspan="5">Research hours
0-5 mins
5-30 mins
30 mins-1 hr
1-4 hrs
4-8 hrs</td></tr>
<tr><td colspan="2"></td></tr>
<tr><td colspan="2"></td></tr>
<tr><td colspan="2"></td></tr>
<tr><td colspan="2"></td></tr>
</table>
